# Severe Dengue With Hyperinflammatory State and Associated Acute Pancreatitis

**DOI:** 10.1155/crdi/8029446

**Published:** 2025-04-12

**Authors:** Susan Alessandra Rodriguez Gonzalez, Linda Banegas, Edwin Mauricio Cantillano Quintero, Jesús Domínguez-Rojas

**Affiliations:** ^1^Departamento de Pediatria, UNAH-VS Universidad Nacional Autónoma de Honduras, Valle de Sula, Tegucigalpa, Honduras; ^2^Departamento de Cuidados Intensivos Pediátricos, Hospital Regional del Norte, Instituto Hondureño de Seguridad Social, San Pedro Sula, Honduras; ^3^Departamento de Emergencias y Áreas Críticas, Instituto Nacional de Salud del Niño, Lima, Peru

**Keywords:** acute pancreatitis, cytokine release syndrome, severe dengue

## Abstract

The increase in the incidence of dengue cases in the region has led to the identification of multiple complications associated with the disease. The present study presents the case of an adolescent girl with severe dengue in a hyperinflammatory state, who presented mild acute pancreatitis. This complication, although infrequent, can be harmful. In the clinical case presented, the patient presented severe dengue, according to the clinical diagnosis. In addition, polyserositis was observed, with positive NS1, IgG, and IgM, elevated transaminases above 1000 U/L and elevated levels of inflammatory markers such as ferritin, LDH, procalcitonin, and decreased fibrinogen. The diagnosis of acute pancreatitis was established based on two main criteria: elevated serum lipase/amylase and imaging studies. The patient did not experience significant abdominal pain; however, she manifested intense and persistent nausea for a period of 3 days. Despite presenting no other risk factors for acute pancreatitis, the patient was suffering from an infectious process. Treatment consisted of a complete 24-h fast and adequate hydration, which resulted in a progressive improvement in the patient's condition.

## 1. Introduction

The global incidence of dengue has increased considerably over the past 2 decades, both globally and in the Americas region, where 80% of the world's cases are registered [1].

The disease may manifest in a range of forms, from asymptomatic to severe and life-threatening cases. Such manifestations may include shock due to plasma loss, fluid accumulation, respiratory distress, severe bleeding, and organ dysfunction. Acute pancreatitis represents an uncommon manifestation of the disease. Some published studies have reported an incidence of acute pancreatitis of 1.33% [2]. The enlarged pancreas was found in 41 patients (29%), 10 (14%) of whom had mild dengue hemorrhagic fever and 31 (44%) of whom had severe dengue hemorrhagic fever in the Setiawan et al.'s study [3].

The etiology and pathogenesis of acute pancreatitis in viral infections remain unclear. A number of hypotheses have been put forth, including the potential involvement of diffuse edema within the interlobular septum due to vascular permeability, as observed in histopathological studies of two fatal cases in Brazil [4]. Other hypotheses posit that viral infection elicits an autoimmune response to pancreatic islet cells, resulting in edema formation in the ampulla of Vater and subsequent pancreatic fluid outflow obstruction. In addition, the direct destruction and inflammation of pancreatic acinar cells by the same virus have been proposed as potential etiologies [2].

In light of the prevailing epidemiological circumstances, characterized by an increase in the incidence of cases, the knowledge of this particular complication, although infrequent, enables earlier intervention with patients, which may result in a more favorable outcome.

## 2. Case Report

A 17-year-old adolescent was admitted to the hospital with persistent fever for four days. On admission to the emergency department, he tested positive for NS1, IgG, and IgM antibodies to dengue, with a hematocrit of 42.3% and a platelet count of 19,000 mm^3^. In addition, his initial vital functions were as follows: heart rate 120 bpm, respiratory rate 20 rpm, temperature 36°C, and oximetry 96% in room air. He presented symptoms such as nausea, polyserositis, ascites, and edema in arms and legs. The clinical and serological diagnosis was established based on the presence of alarm signs, and positivity for NS1, IgG, and IgM for dengue, as well as other indicators of inflammation, such as elevated levels of ferritin, lactate dehydrogenase, procalcitonin, and fibrinogen. The patient's serum bilirubin level was 164 mg/dL, aspartate aminotransferase (AST) was 2826 units/L, alanine aminotransferase (ALT) was 1290 units/L, lipase was 105 units/L, and pancreatic amylase was 91 units/L. Abdominal ultrasound revealed a pancreas of typical dimensions and morphology, with a length of 2 cm in head, body, and tail.

**Figure 1:**
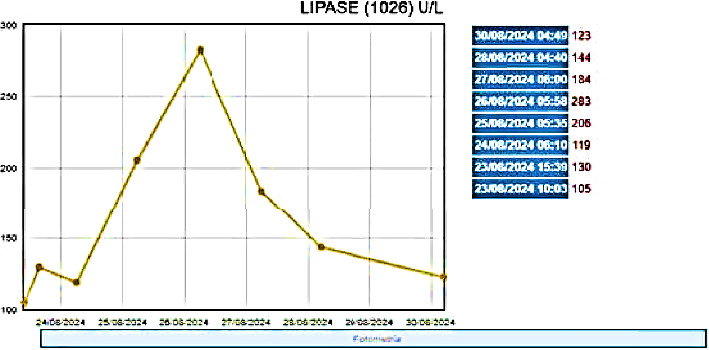
Serum lipase levels graph.

She was admitted to the pediatric intensive care unit for treatment of severe dengue, with intravenous fluid support and methylprednisolone at 1 mg per kilo per day. During her stay, the patient continued to experience nausea and anorexia for two days, with no abdominal pain and with an increase in lipase levels to 283 μ/L (reference value: 13–60 μ/L) (Figure 1). In view of this situation, a new abdominal ultrasound was performed on the seventh day of the disease, which revealed a hypoechogenic pancreas, with a head of 1.2 cm, a body of 1.1 cm, and a tail of 2 cm. A thoracic ultrasound was performed showing slight bilateral effusion and scarce liquid content in the pericardium in cardiac ultrasound.

**Figure 2:**
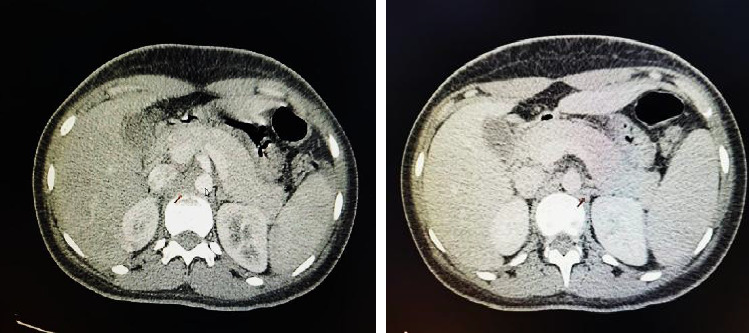
Abdominal CT images in arterial and venous phase showing edematous pancreas and inflammatory changes of the peripancreatic fat.

The study performed by contrast-enhanced abdominal CT revealed the presence of an edematous pancreas and scarce free fluid, accompanied by inflammatory changes in the peripancreatic fat. However, no signs of necrosis, hemorrhage, or collections were observed (Figure 2). The patient did not present clinical manifestations suggestive of gallstones nor did she have a history of alcohol consumption, hypertriglyceridemia, trauma, or drug use. The only associated process was infectious in nature. The patient underwent a 24-h fast, after which a defatted oral diet was started, resulting in an effective treatment.

## 3. Discussion

Acute pancreatitis is a serious pathology which, under certain circumstances, can be lethal. Its incidence in the pediatric population is significantly lower than in the adult population [5], while dengue fever is a disease that can affect multiple organs and present with atypical symptoms. The World Health Organization (WHO) has established the expanded dengue syndrome (DES) [6] as a category that encompasses these conditions, which can compromise the functionality of multiple organs and generate complications such as acute pancreatitis. Despite the low prevalence of DES, it is imperative to consider this diagnosis for timely identification and implementation of specific treatments. In this regard, it has been observed that the use of corticosteroids in patients with expanded dengue can suppress cells involved in innate immunity, such as T cells, B cells, and antibodies, complements, and hematological manifestations in dengue pathology [7].

However, few studies have been conducted to evaluate the efficacy of corticosteroids in dengue infection, although the immunologic pathology of dengue is analogous to that of other diseases effectively treated with corticosteroids for several decades. For the diagnosis of acute pancreatitis in children, the diagnostic criteria proposed by International Study Group of Pediatric Pancreatitis: In Search for a Cure (INSPPIRE) are a valuable resource. The presence of at least two of the following symptoms should raise suspicion of acute pancreatitis: abdominal pain compatible with or suggestive of pancreatitis, elevated serum lipase or amylase at least three times the upper limit (expressed in IU/mL), and imaging findings compatible with pancreatitis [6].

Abdominal discomfort is considered a frequent symptom of dengue, although it is rarely associated with this complication. Therefore, it is crucial to consider such a possibility, especially if the discomfort persists beyond the critical phase. Although the patient did not present with the typical abdominal pain associated with this condition, the persistence of nausea led to the formulation of a clinical suspicion, which was later confirmed by complementary studies.

A systematic review studying 9365 dengue patients found that 16% of the patients presented with acute abdomen; 7.7% of which were attributed to acute pancreatitis [8].

The clinical evolution of our patient was favorable during his hospital stay thanks to timely detection and early treatment, and he was discharged home.

It is imperative to perform laboratory and imaging studies in case of complications, especially if the expected evolution differs from the usual.

## 4. Conclusion

In conclusion, although acute pancreatitis in the context of dengue is not a frequent manifestation, its occurrence represents an important clinical challenge.

It is imperative to deepen in the possible mechanisms of interaction between dengue and pancreatitis to facilitate a better diagnosis, management, and prevention of this complication.

## Data Availability

The data used to support the findings of this study are available from the corresponding author upon reasonable request.
